# Roads to Stat3 Paved with Cadherins

**DOI:** 10.3390/cells11162537

**Published:** 2022-08-16

**Authors:** Hanad Adan, Juliet Daniel, Leda Raptis

**Affiliations:** 1 Department of Biology, McMaster University, Hamilton, ON L8S 4L8, Canada; 2 Department of Pathology and Molecular Medicine, Queen’s University, Kingston, ON K7L 3N6, Canada

**Keywords:** Stat3, cadherins, gp130, IL6, rac1, Src, caveolin-1, FAK, cell density

## Abstract

The engagement of cadherins, cell-to-cell adhesion proteins, triggers a dramatic increase in the levels and activity of the Rac/Cdc42 GTPases, through the inhibition of proteasomal degradation. This leads to an increase in transcription and secretion of IL6 family cytokines, activation of their common receptor, gp130, in an autocrine manner and phosphorylation of the signal transducer and activator of transcription-3 (Stat3) on tyrosine-705 by the Jak kinases. Stat3 subsequently dimerizes, migrates to the nucleus and activates the transcription of genes involved in cell division and survival. The Src oncogene also increases Rac levels, leading to secretion of IL6 family cytokines and gp130 activation, which triggers a Stat3-ptyr705 increase. Interestingly, at the same time, Src downregulates cadherins in a quantitative manner, while cadherins are required to preserve gp130 levels for IL6 family signalling. Therefore, a fine balance between Src^527F^/Rac/IL6 and Src^527F^/cadherin/gp130 levels is in existence, which is required for Stat3 activation. This further demonstrates the important role of cadherins in the activation of Stat3, through preservation of gp130 function. Conversely, the absence of cadherin engagement correlates with low Stat3 activity: In sparsely growing cells, both gp130 and Stat3-ptyr705 levels are very low, despite the fact that cSrc is active in the FAK (focal adhesion kinase)/cSrc complex, which further indicates that the *engagement* of cadherins is important for Stat3 activation, not just their presence. Furthermore, the caveolin-1 protein downregulates Stat3 through binding and sequestration of cadherins to the scaffolding domain of caveolin-1. We hypothesize that the cadherins/Rac/gp130 axis may be a conserved pathway to Stat3 activation in a number of systems. This fact could have significant implications in Stat3 biology, as well as in drug testing and development.

## 1. Introduction

Cell culture techniques developed in the last 80 years have been instrumental in the investigation of cellular processes. Adherent cells grow while attached to a plastic petri dish surface till contact inhibition of growth is reached at confluence. Invariably, cells are studied at the time when they are actively growing, that is, at subconfluence. However, in normal tissues or tumors in vivo, cells have extensive opportunities for adhesion to their neighbors (unlike sparsely growing, cultured adherent cells), while the mechanical forces applied upon each cell in a three-dimensional tissue architecture are important determinants of cellular functions such as proliferation, differentiation or apoptosis [1]. However, confluent cell cultures, although two-dimensional, may in part mimic some of the physiological stress signals of tissues.

In this communication, we integrate some recent findings regarding a common signal transducer, the signal transducer and activator of transcription-3 (Stat3), which undergoes a dramatic surge in activity following cell-to-cell contact mediated by the cadherin class of adhesion receptors, as occurs with the confluence of cultured cells. Contrary to accepted dogma, even oncogenes such as Src that downregulate cadherins still require some cadherin action to activate Stat3. These findings bring to the fore the relevance of observations made in cultured cells grown to high densities to the situation in vivo.

## 2. Cell-to-Cell Adhesion Triggers a Dramatic Increase in the Levels of Rac and IL6, Leading to Stat3 Activation

### 2.1. The Signal Transducer and Activator of Transcription-3 (Stat3)

The signal transducer and activator of transcription family of proteins (STATs, Stat1 to Stat6) were initially discovered as important mediators of cytokine signaling (reviewed in [2]). Later studies revealed that certain STATs such as Stat3 are also activated by receptor and nonreceptor tyrosine kinases (RTKs and NRTKs) such as the epidermal growth factor or platelet-derived growth factor receptors (EGFR or PDGFR [3]) and Src [4,5,6].

Like other STATs, Stat3 is latent in the cytoplasm in unstimulated cells. Receptor activation following ligand engagement leads to phosphorylation of a specific phosphotyrosine of the receptor, which offers a docking site where Stat3 binds through its SH2 (src-homology 2) domain. Stat3 is then phosphorylated in the receptor complex by the receptor itself or by associated Src or Jak kinases, at a critical tyrosine (tyr705) [7,8]. This phosphorylation leads to dimerization through reciprocal SH2-ptyr705 interactions between two molecules, which trigger Stat3′s nuclear translocation and binding to specific DNA sequences (TTC-NNN-GAA) to initiate the transcription of a number of genes [9]. Stat3-responsive promotors include genes involved in cell division, such as *myc,* the hepatocyte growth factor receptor [10], cyclin D1 and E, as well as the cyclin-dependent kinase inhibitor, p21^CIP/WAF^ [11], or survival, such as *bcl-xL*, *mcl-1* and *survivin* [12,13], and the oxygen sensor HIF1α (hypoxia-inducible factor-1α) transcription factor [14], while it downregulates p53 [15]. Interestingly, Stat3 can also provide a survival signal by affecting the mitochondria through its ser 727 phosphorylated form, which occurs through MAP kinase pathways, such as Ras signaling or stress [16,17,18,19,20].

Stat3 is hyperactive in a number of human cancers [13], and the fact that Stat3C, a constitutively active form of Stat3, is sufficient to induce neoplastic transformation of nontransformed mouse fibroblasts [21] points to an etiological role for Stat3 in such tumors.

### 2.2. The Cadherin Family of Cell-to-Cell Adhesion Receptors

Cell-to-cell adhesion is mediated by the cadherin class, calcium-dependent adhesion receptors [22]. Classical cadherins are plasma membrane glycoproteins that control the organization, specificity and dynamics of cell adhesion, which is crucial for the development and maintenance of tissue architecture and function [23,24]. Classical cadherins comprise an extracellular or ecto-domain, a single-pass transmembrane domain and an intracellular domain, which interacts with the cytoskeleton. The ectodomain consists of five modules (EC1 to EC5) of approximately 100 amino acids each with internal sequence homology [25]. The extracellular segments present on the surface of opposing cells interact in a homophilic manner to create highly regulated patterns of attachment, stabilized by cytoskeletal elements inside the cells. The adhesive interface is exclusively on the outward-most EC1 module. Cadherin engagement initiates intracellular signals regarding cytoskeletal organization, cell polarity, proliferation or apoptosis that are communicated through the conserved cadherin tail domain to different cytoplasmic pathways [26,27]. Five members of the type I (e.g., E-cadherin) and twelve members of the type II (e.g., cadherin-11, Cad11), classical cadherin subfamilies that are expressed in different tissues and may play a role in metastasis have been identified [28,29,30,31].

### 2.3. Cadherin Engagement, Rac, IL6 and Stat3 Activation in Nonneoplastic Cells

As often happens, serendipity led us to the discovery that the confluence of cultured cells triggers a dramatic increase in the activity of Stat3; in attempting to examine whether a viral, nuclear oncogene, the simian virus 40 large tumor antigen (SVLT), which was previously shown to activate the Ras pathway [32], is able to activate Stat3 upon expression in established rodent fibroblasts, we discovered that although SVLT does activate Stat3 [33], cell density per se also confers a dramatic increase in Stat3 activity [34]. Given the fact that Stat3 is known to be hyperactive in a number of tumors, in tumor-derived cell lines as well as in cell lines transformed by a variety of oncogenes, it was expected that confluence-induced growth arrest would suppress Stat3 activity. The dramatic increase in the cells’ Stat3 activity at several days after confluence was a highly unexpected finding; therefore, its incidence and mechanism were investigated further.

**Figure 1:**
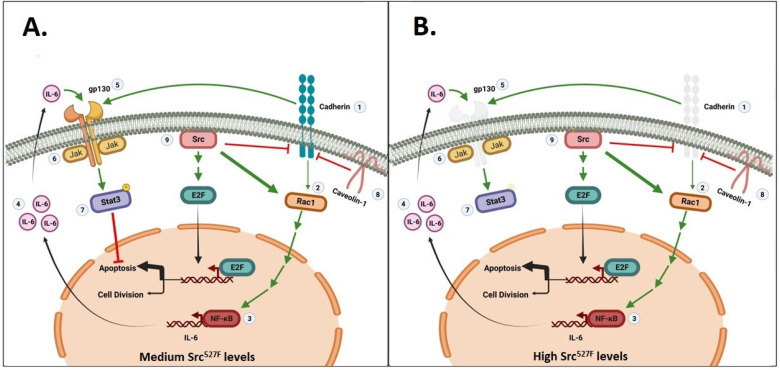
The engagement of cadherins from opposing cells increases Rac and Stat3 activity and is required for Src-mediated Stat3 activation and cellular survival (data from [48]). Engagement of cadherins (1) increases Rac (2) levels and activity, leading to Stat3 (7) activation through NFκB (3), IL6 (4), gp130 (5) and Jak (6). Caveolin-1 (8) reduces gp130 and Stat3-ptyr705 by binding to and sequestering cadherins into its scaffolding domain [50]. Src^527F^ (9) also increases Rac levels and activity, independent from cadherin engagement (see Figure 3A in Ref. [48]). What follows, and its effect upon Stat3, depends upon the levels of Src^527F^ expression: (**A**) medium Src^527F^ levels: activation of Rac leads to the secretion of IL6 family cytokines and the activation of gp130, Jak and Stat3 (see Figure 2B in Ref. [48]). At the same time, gp130 function requires cadherin engagement, but cadherins are downregulated by Src^527F^. However, at medium Src^527F^ levels there is enough residual cadherin/gp130 so as to allow Stat3-ptyr-705 phosphorylation and activation (adapted from [45,46,47,48]). (**B**) High Src^527F^ levels: cadherin levels are reduced to nondetectable, and this leads to gp130 downregulation (see Figure 5A,B in Ref. [48]), with a dramatic reduction in Jak and Stat3 activities as a result (see Figure 2 in Ref. [48]). Generated in BioRender.

Examination of Stat3-ptyr705 levels of cultured cells, from 50% confluence up to 1–4 days after confluence (depending upon the cells’ growth rate), revealed a dramatic increase in a large number of breast [34] or lung [35,36,37] cell lines, as well as in nontransformed human breast MCF10A [34], nontransformed mouse epithelial HC11 cells [38] and fibroblasts such as Balb/c3T3, before (Figure 1) or after expression of SVLT [33]. The results from other labs showed a similar increase as cells reached confluence ([39,40,41,42], reviewed in [43]). This increase was found to require Jak but to be independent from a number of factors known to be involved in cell adhesion signaling, such as the Src family (Src, Fyn, Yes), Fer, IGF1-R, EGFR and Ras [34]. The fact that calcium chelation reduced, while cell aggregation dramatically increased Stat3-ptyr705 pointed to a cadherin involvement [34]. This was further shown by the fact that peptides encompassing the HAV domain of cadherins that line the adhesion surface (SHAVSA for E-cadherin in epithelial cells [44,45]) could reduce Stat3-ptyr705 in confluent cultures. Cadherin involvement was definitively demonstrated by a dramatic increase in Stat3-ptyr705 following plating mouse breast epithelial HC11 cells on plastic petri dish surfaces functionalized with a cloned fragment encompassing the two outermost domains of E-cadherin, or plating mouse Balb/c3T3 fibroblasts on the corresponding fragment of Cad11, respectively [45,46].

Further examination of the mechanism of the cadherin-mediated Stat3 activation revealed that E-cadherin engagement triggers a dramatic increase in the levels and activity of Rac and Cdc42, small GTPases in HC11 cells [45], through inhibition of proteasomal degradation [47]. This leads to an increase in transcription of the interleukin-6 (IL6) family cytokines, which bind to their receptor in an autocrine manner to activate Stat3 through the Jak kinases [45]. A similar mechanism of Stat3 activation by Cad11 through Rac and IL6 was also described [46]. In addition, the expression of mutationally activated Rac^V12^ triggered Stat3 activation in both epithelial cells and fibroblasts, through the autocrine secretion of IL6 family cytokines [47], further reinforcing the concept that IL6 secretion may be part of the pathway of cadherins to Stat3. Interestingly, cadherin engagement also increases the stability of receptors such as the gp130 subunit of the IL6 receptor family, thus increasing Stat3-ptyr705 further [48] (Figure 1).

The role of Stat3-ptyr705 following cadherin engagement in cells growing to high densities was examined through inhibition experiments. The treatment of confluent cultures with the Stat3-selective CPA7 inhibitor, expression of the dominant-negative mutant Stat3β or expression of an antisense construct triggered apoptosis [34], indicating that Stat3 offers a potent survival signal in this setting [33,49]. The importance of survival in confluent cultures, or in normal tissues in vivo, may be to counteract the apoptosis created by mechanical constraints that may reduce integrin adhesion to the extracellular matrix and F-actin formation. This would allow the activation of LATS (large tumor suppressor) and hence inactivation of the transcriptional coactivators YAP/TAZ and apoptosis [1,18]. In any event, the fact that the cell is calling up Stat3 rather than PI3k (phosphatidyl-inositol-3 kinase) from latency may be an indication that Stat3 is a more powerful survival signal compared to PI3k.

Stat3 plays a critical role in cell differentiation as well: it has long been established that confluence controls the differentiation of mouse HC11 nontransformed breast epithelial cells, and it turned out that it is due to cadherin engagement [38]. In fact, confluence and the addition of prolactin, insulin and hydrocortisone to HC11 cells trigger an increase in cRac and Stat3-ptyr705, which are necessary for differentiation. Interestingly, while the expression of low levels of mutationally activated Rac^V12^ dramatically increased differentiation, the expression of high Rac^V12^ levels blocked differentiation concomitant with E-cadherin downregulation, while inducing neoplastic transformation [38].

## 3. Activated Src^527F^ Requires Rac, Cadherins and gp130 to Activate Stat3

### 3.1. The Src Oncogene and the Polyoma Virus Middle Tumor Antigen

The Src family of NRTKs is often hyperactive in a variety of cancers [51], while activated Src is frequently associated with worse patient survival [52]. The cellular homolog of the viral Src, c-Src, is held in an inactive state through the binding of a ptyr on its carboxy-terminus (ptyr527) to an SH2 domain on the Src amino-terminus. Mutation to 527F forces an open configuration and renders the molecule active regarding its kinase activity as well as neoplastic transformation [53].

c-Src can also be activated by the middle tumor antigen of polyoma virus (mT). Poly-oma virus transformation is driven mainly via its mT, an oncogene that binds the carboxy-terminus of c-Src and increases its kinase activity [54,55]. c-Src then phosphorylates mT at distinct tyrosine residues where cytoplasmic signal transducers bind with their cognate SH2 (Src-homology-2) or PTB (phosphotyrosine-binding) domains (ptyr315: PI3k/Akt, ptyr250: Grb2/Ras, ptyr322: PLCγ) [56,57,58,59,60,61,62,63,64], leading to anchorage-independent proliferation and tumorigenicity [65,66]. Thus, following phosphorylation by activated c-Src, mT acts as a scaffold to activate several signal transducers, leading to full neoplastic conversion.

### 3.2. Stat3 Activation by Src^527F^ Requires Cadherin-11

**Figure 2:**
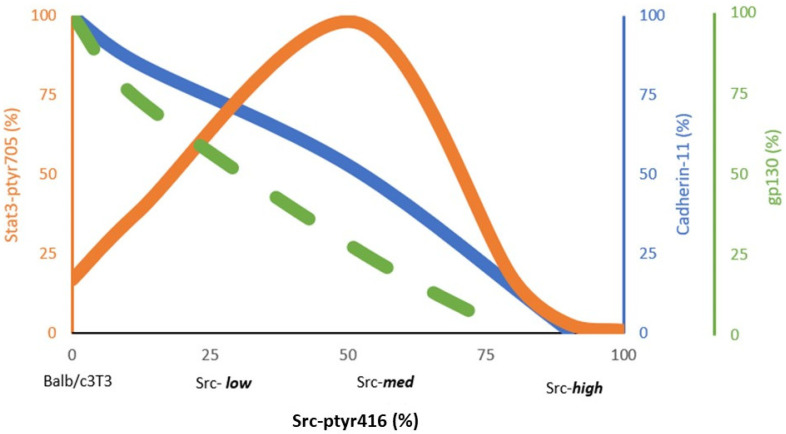
Cadherin-11, Stat3-ptyr705 and gp130 levels relative to Src^527F^. Relative levels of Stat3-ptyr705, cadherin-11 and gp130 were assessed by Western blotting in mouse Balb/c3T3 fibroblasts expressing different amounts of Src^527F^, with the highest taken as 100%. Note that Cadherin-11 and gp130 are reduced with increasing Src^527F^, while Stat3-ptyr705 is highest at Src^527F^ levels of approximately 50% and is dramatically reduced at high Src^527F^ levels. Peak values regarding the cell density of a number of cell lines are shown [48]. Technical detail: Uniform distribution and low cell-to-cell contact at plating is very important. Cells must be passed from a subconfluent petri and vigorously pipetted with a 9 inch pasteur pipette.

Activated Src^527F^ negatively regulates E-cadherin function in epithelial cells (reviewed in [67]), as well as Cad11 levels in mouse or rat fibroblasts [48]. Furthermore, by expressing different Src^527F^ levels, a quantitative, inverse relationship between Src^pY416^ and Cad11 levels was recently discovered; cells expressing the highest Src^527F^ levels (Src-high clones) had no detectable Cad11, while lines expressing 50% of Src^527F^ had approximately 42% (Src-med) and cells expressing 25% of Src^527F^ had 75% (Src-low) the Cad11 protein levels of the parental Balb/c3T3 cells [48] (Figure 2).

Early results demonstrated that activated Src expression in cultured cells increases Stat3-ptyr705 levels, DNA binding and transcriptional activity. Furthermore, Stat3 activity was found to be required for neoplastic transformation by the Src oncogene [4,5]. However, despite the well-established notion that Src activates Stat3, we recently discovered that Stat3 activation is highly dependent upon levels of Src^527F^ expression: Src^527F^ expression to low (25% of the highest) or intermediate (50% of the highest) levels increased Stat3-ptyr705, as previously documented [4,5] (Figure 2). Interestingly however, there was a complete absence of detectable Stat3-ptyr705 (even lower than the parental Balb/c3T3), at high Src expression levels when Cad11 was absent as well [48] (Figure 1B and Figure 2). This was confirmed in rat-F111 fibroblasts transformed through the expression of high amounts of mT [48,66]. Furthermore, the expression of Src^527F^ in mouse fibroblasts rendered Cad11-deficient through stable expression of an shCad11 antisense construct (shCad11 cells) was unable to trigger an increase in Stat3-ptyr705 levels, indicating that Src^527F^ requires Cad11 in order to activate Stat3; in the absence of Cad11, either due to expression of high Src^527F^ or expression of shCad11, Stat3-ptyr705 levels were undetectable [48].

### 3.3. Cadherins Are Required for gp130 Receptor Function and Stat3-ptyr705 Phosphorylation

The Rac GTPase was shown to be required for Stat3 activation by Src through an effect upon the Tiam GTP/GDP exchange factor [68], but also through an increase in total Rac protein [48]. Rac subsequently triggers the secretion of IL6 family cytokines that, in turn, activate their cognate receptor, gp130 [47]. Interestingly, cells expressing high Src^527F^ also secrete high amounts of Stat3-activating cytokines. However, although these cytokines are able to stimulate Stat3-ptyr705 phosphorylation in the parental Balb/c3T3 cells if added to their medium, these cytokines are not able to activate Stat3 in the cells secreting them, in an autocrine manner [48]. This was demonstrated to be due to the fact that Src-high cells lack gp130 receptors [48]. Therefore, it appears that Cad11 is required to stabilize the gp130 receptor. This conclusion is reinforced by the fact that shCad11 cells were shown to lack gp130 as well [48], and is consistent with the E-cadherin requirement for proper activation of the gp130 receptor in mouse embryonic stem cells [69]. Since Src-high cells appeared to be completely devoid of Stat3-ptyr705, it is likely that receptors other than gp130 that are known Stat3 activators may require Cad11 for maintenance. Therefore, when Src is expressed to intermediate levels, there is enough residual cadherin-11 left to protect gp130 and allow Jak and Stat3 signaling, as expected. Interestingly however, at high Src^527F^ levels, Cad11 is eliminated, hence gp130 signaling and Jak/Stat3 activation is abolished. That is, a fine balance between Src/Rac/IL6 and Cad11/gp130 is required so as to induce the secretion of IL6 family cytokines (through Rac) and preserve sufficient gp130 levels (through Cad11) to allow signalling through the Src/Rac/IL6/gp130/Jak/Stat3 axis (Figure 1).

In addition to Stat3, Src^527F^ is known to activate the E2F transcription factor family, which promotes apoptosis through the Ras/Raf/Erk pathway [70]. The fact that cells with intermediate Src levels and high Stat3 displayed a greater degree of apoptosis (assessed by PARP cleavage and TUNEL staining) compared to the parental Balb/c3T3 shows that the pro-apoptotic effect of E2F activation by Src^527F^ may be prevailing over the survival effect of Stat3 [48] (Figure 1). Therefore, the Cad11/Rac/Stat3 axis is emerging as a crucial signalling pathway for the survival of cells transformed by Src^527F^ and perhaps other oncogenes.

## 4. Sparsely Growing, Nontransformed Cells: No Cadherin Engagement, No gp130, No Stat3-ptyr705

When adherent cells are cultured to high densities while attached to a plastic petri surface, the activated Stat3 offers a potent survival signal. Conversely, when cells are grown sparsely, their opportunities for cell-to-cell adhesion through cadherin engagement are minimal or absent, hence Stat3 activity is very low, further confirming the importance of cadherin engagement in Stat3 activation. However, the question remains: What offers survival signals to sparsely growing cells?

At low densities, as the cells are extended, cell functions are maintained through cellular anchoring to the underlying extracellular matrix (ECM) substratum through integrins (β1 and β4 subtypes) and cytoskeletal signaling [71,72]. Engagement of integrins with the focal adhesion kinase (FAK) activates the FAK/cSrc complex, and this results in the activation of a number of tyrosine kinase receptors, Erk1/2 and Akt-pser473 [73,74], as shown through extensive inhibition experiments. Interestingly, despite the ability of the integrin/FAK/cSrc complex to activate Erk and Akt, the complex, or the receptors activated by it, cannot activate Stat3 in sparsely growing cells, i.e., in the absence of Cad11 engagement [74]. Since at low densities gp130 is also very low, it appears that cadherin engagement from the surfaces of opposing cells, not simply cadherin expression, is required to maintain gp130 function. This is despite the fact that the FAK/cSrc kinase complex strongly activates Erk1/2 and Akt. These findings further stress the importance of cadherin engagement for Stat3 activation. As a result of Akt and Erk1/2 activation by FAK/cSrc, Akt-pser473 and p-Erk1/2 levels are high at all densities of attached cells [73].

## 5. Caveolin-1 Reduces Stat3-ptyr705 through Cadherin Downregulation

The Cad11 requirement for Src^527F^ to activate Stat3, granted by Cad11′s ability to preserve gp130 integrity, is one example of the importance of cadherins in Stat3 activation. Interestingly, Cad11 is also the vehicle for Stat3′s downregulation by another membrane protein, caveolin-1 (Cav1), that participates in the membrane signaling apparatus.

### 5.1. Caveolae and Caveolins

Caveolae are cholesterol-rich, 50–100 nm invaginations of the plasma membrane whose role is the transport of materials and cholesterol homeostasis, as well as signal transduction. Caveolins (Cav1–3) are embedded in the lipid bilayer of caveolae and are considered to be their marker proteins [75,76,77].

The role of Cav1 in signal transduction is complex [78]. Cav1 sequesters, hence inactivates, membrane signaling molecules such as a large number of RTKs and components of signaling pathways such as the epidermal growth factor receptor (EGFR) family, Ras, Raf, Mek, Erk, PI3k and others, which bind to the scaffolding domain (CSD) of caveolins [75,79,80,81]. However, other receptors such as the insulin receptor [82] are positively regulated through Cav1 interaction (reviewed in [78]).

### 5.2. Cav1 Negatively Regulates Stat3 through Cadherin-11 Downregulation in Mouse Fibroblasts and Lung Cancer Lines

Data from transgenic animals or tumors revealed a ***negative*** role of Cav1 upon Stat3-ptyr705; lung tissues from Cav1 knockout mice displayed high Stat3-ptyr705 levels [83], while Cav1-KO mice were more susceptible to tumorigenesis by a variety of oncogenes [84]. In addition, in normal human and mouse breast tissues, high Cav1 correlated with low Stat3-ptyr705 [45,85], reinforcing the notion of a negative effect. Examination of the Cav1/Stat3 relationship in cell culture experiments demonstrated that stable Cav1 overexpression with a retroviral vector reduced Stat3-ptyr705 levels and, as expected, promoted apoptosis in mouse fibroblasts and in lung carcinoma SHP77 cells [50]. Conversely, stable downregulation of Cav1 through shRNA expression with a retroviral vector resulted in an increase in Stat3-ptyr705 [50]. Most importantly, Cav1 overexpression also triggered a dramatic reduction in Cad11 and Rac levels in SHP77 cells, a process that required the CSD domain of Cav1, while Cav1 knockdown increased Cad11 levels [50]. Taken together, the above data demonstrate that Cav1 downregulates Stat3 through Cad11 downregulation by a process requiring the CSD domain of Cav1. This further stresses the power of cadherins towards Stat3 activation [50] (Figure 1).

## 6. Discussion

Cell-to-cell adhesion through the engagement of cadherins brings about seismic changes to certain cellular processes. Here, we integrate evidence demonstrating the key role of cadherins upon activation of Stat3, in both nontransformed and neoplastic cells. The following questions are of utmost importance:

1. What is the role of cadherins in the activation of Stat3?
2. What are the implications of the cadherin-mediated activation of Stat3 for drug development?

### 6.1. Role of Cadherins in Stat3 Activation

Following their discovery, the cadherin family of proteins were assumed to play a primarily structural role of cell-to-cell adhesion to form a tissue [86]. However, it was later demonstrated that cell-to-cell adhesion leads to Stat3 activation, without the exogenous expression of oncogenes (reviewed in [18,43]). In fact, cadherin engagement triggers a dramatic increase in the levels and activity of Rac through inhibition of proteasomal degradation [45,47]. This unleashes a sequence of signaling events including the secretion of IL6 and activation of Stat3 in an autocrine manner through the gp130 common receptor. Interestingly, Stat3 activation was found to be independent from cellular forms of the Src family (cSrc/Fyn/Yes), IGF1-R and Fer [34] and to offer a potent survival signal.

Many cytoplasmic signal transducers bind to specific phosphotyrosine sites of growth factor receptors or oncogenes directly, through their SH2 or PTP domains. However, although Stat3 has an SH2 domain, it is not activated through direct binding to oncogenes such as Src. In fact, it became evident early on that, despite the fact that Src is a potent Stat3 activator, Stat3-ptyr705 phosphorylation by Src requires growth factor receptors, acting as mere scaffolds, that is, even if they are devoid of kinase activity [87]. In this model, Src phosphorylates and activates Jak, which in turn phosphorylates the receptor (s). Stat3 subsequently binds with its SH2 domain to the receptor and is phosphorylated by Src. Later data [48] from Src^527F^-expressing, mouse Balb/c3T3 fibroblasts further revealed that Src itself (independent of cadherin engagement) increases Rac levels and activity, which activates Stat3 through the IL6/gp130/Jak pathway (Figure 1). In this schema, cadherins (Cad11, and also perhaps E-cadherin and N-cadherin [46]) play a crucial role in preserving the function of gp130 [69], which is key for Jak binding to allow Stat3 activation by Src. Given the established notion that Src effectively downregulates E-cadherin [67] and Cad11 function [48], the demonstration that Src does require cadherin action to activate Stat3 was a highly unexpected finding. In fact, high Src expression eliminates cadherins, hence gp130, with a dramatic reduction of Stat3-ptyr705 to undetectable levels as a result. Furthermore, the fact that high Src completely eliminates Stat3 points to the possibility that, in addition to gp130, Src may destroy other receptors as well, such as EGFR and PDGFR [88], which are known Stat3 activators [3], and these can be rescued by resi-dual cadherin action.

A similar requirement for gp130 was seen in Stat3 activation by the mT oncogene of polyoma virus. mT normally binds to and activates cSrc, which, in turn, phosphorylates distinct phosphotyrosine sites on mT, which become docking sites for cytoplasmic signal transducers. Thus, although mT is normally acting as a scaffold, it still requires the gp130 scaffold for Jak binding and Stat3 activation, which stresses the role of cadherins in maintaining gp130 levels for Stat3 activation to occur [48]. Since Src increases Rac levels and activity, it would be expected that mutationally activated Rac^V12^ and perhaps other oncogenes would require cadherins and gp130 for neoplastic transformation.

The importance of cadherins for Stat3 activation is also shown in instances of Stat3 downregulation: In sparsely growing cells, both gp130 and Stat3-ptyr705 levels are very low, despite the fact that cSrc is active in the FAK/cSrc complex. This further indicates that the engagement of cadherins is important, not just their expression levels, which change only marginally with confluence. In this case survival signals are offered by the integrins/FAK/cSrc complex that activates Akt, thus preventing apoptotic death of single cells that lack cell–cell contacts [73]. Similarly, caveolin-1, through its scaffolding domain, may sequester cadherins to reduce Stat3 activity. As a result, caveolin-1 overexpression triggers Stat3 downregulation and apoptosis [50].

### 6.2. Stat3 as a Cancer Therapy Target—Design and Testing of Inhibitors

The majority of tumors display high levels of activated E2F transcription factor family (E2F1-3a [89]), as a result of phosphorylation and inactivation of the Rb (retinoblastoma susceptibility) family of nuclear phosphoproteins by the Ras/Raf/Mek/Erk pathway, which is activated by Src family kinases or activated membrane receptors. E2F targets genes involved in DNA synthesis, and growth factor and receptor genes [90]. Interestingly, at the same time, E2F activates apoptosis genes [91,92], but apoptosis is prevented due to the activation of survival factors such as PI3k and Stat3 by tyrosine kinase receptors induced by E2F itself, or directly by Src or other kinases, so that transformation does occur (Figure 1). As a result, upon inhibition of Stat3, tumour cells, having high E2F levels, will succumb to apoptosis [33]. Interestingly, in tumours where Cad11 or N-cadherin promotes metastasis (e.g., breast and prostate cancers, [93,94,95,96,97,98]), then inhibition of Cad11 would induce apoptosis (through Stat3 inhibition) in metastatic tumour cells specifically, since normal cells would have low E2F activity, hence would be spared. On the other hand, inhibition of Src itself would simply reverse transformation without death of the tumor cell. The therapeutic implications of this observation have attracted attention as a target for cancer therapy, and several Stat3 inhibitors have been designed [99].

Following initial screening by the addition of the compounds to cell extracts directly, testing of new Stat3 inhibitors is invariably conducted by the treatment of cultured cells expressing Src or other Stat3-activating oncogenes, or in cells stimulated with Stat3-activating growth factors or cytokines. Experiments are invariably conducted on subconfluent, i.e., actively growing, cells, and Stat3 activity is assessed in detergent cell lysates. However, given that confluence causes a dramatic increase in Stat3 activity, it is important to take cadherin engagement into account for the following reasons:

1. Factors that promote cadherin disengagement, such as calcium chelators [34] or HAV peptides [45], would also rapidly reduce Jak and Stat3 activity (within 15–60 min [34]), with no effect upon Stat3 per se. The same would hold true with inhibitors of Rac, or IL6 family cytokines, or NFκB. In our hands, even changing the medium of HC11 mouse breast epithelial or Balb/c3T3 cells with calcium-free DMEM eliminated cadherin engagement and dramatically reduced Stat3-ptyr705 within 30–60 min, depending upon cell density (Raptis et al., unpublished).
2. If an inhibitor causes growth retardation by any (unrelated) mechanism, then following treatment for 2–3 days the cells would be less confluent compared to their untreated controls, hence would have lower Stat3-ptyr705 levels due to the lower confluence, not due to a specific inhibition of Stat3 by the compound under study. The dramatic increase in Stat3 activity with cellular confluence dictates that experiments testing Stat3 inhibition must be conducted at a number of densities spanning the peak values of Stat3-ptyr705 (at 1–5 days post 100% confluence, depending upon the cell’s growth rate), and the peaks of treated and untreated cultures compared. The same holds true with experiments dealing with Stat3-activating oncogenes, e.g., the simian virus 40 large tumor antigen [33], adenovirus E1A [100,101], Src [48], or growth factors and cytokines.

The effect of cadherin engagement upon Stat3 activity can also have a profound effect upon the results of commonly used nucleic acid transfection experiments, which may, in turn, affect drug design and testing. In fact, a dramatic increase in Stat3 activity following calcium phosphate transfection was noted, which was evident even in the absence of DNA and was not due to the mere presence of calcium ions [102]. On the other hand, gene expression through electroporation or expression with a retroviral vector did not affect Stat3 activity, while cationic lipids such as Lipofectamine had a less pronounced effect than that of calcium phosphate transfection. Possibly, the presence of the calcium-phosphate precipitate is responsible for an increase in opportunities for cell-to-cell contact and cadherin engagement, which could increase Stat3-ptyr705 for 2–5 days.

### 6.3. Conclusions, Future Directions, and Clinical Relevance

In addition to Src, a large number of oncogenes and growth factor receptors are known Stat3 activators. It is reasonable to speculate that for some of them at least, such as EGFR or oncogenic derivatives, cadherins may play a key role in ensuring receptor function for Stat3 activation ([69,88]). In fact, the existence of lung cancer lines with high Src-ptyr^418^ but low Stat3-ptyr705 [35,37] points to the possibility of Stat3-ptyr705 downregulation by high Src levels, as was demonstrated in mouse fibroblasts exogenously expressing high Src^527F^ levels [48]. In cancers where high Src is the driver, Src inhibition would increase, rather than decrease, Stat3 activity, with a worsening in the clinical picture as a result. This might, in part, explain why Src inhibitors did not perform well in clinical trials [103,104,105,106].

The relevance of the cadherin-Stat3 interactions observed at high cell densities to the in vivo situation was evidenced by the presence of intense immunohistochemical staining for both E-cadherin and Stat3-ptyr705 in normal luminal breast epithelial mouse cells. In sharp contrast, there was little expression of either protein in the surrounding adipose tissue [45]. Regarding tumor cells, the relevance of findings from confluent cultures to the in vivo microenvironment was further stressed by the close correspondence seen between genes expressed in the prostate carcinoma line LNCap when cultured to high, but not low, densities with genes associated with prostate cancer in vivo [107]. We hypothesize that the Stat3 reduction upon expression of high Src activity levels and the concomitant sensitivity to apoptosis may represent an extra regulatory layer to destroy cells with high Src activity for the survival of the organism.

Since the discovery of the Rac/Cdc42 and Stat3 activity surge upon cadherin engagement, other gene products have been shown to be affected by cell density to different degrees, such as Caveolin-1 [50] and connexin-43 [35], while cadherins were shown to be required for gap junctional, intercellular communication [108,109]. Other genes are deemed to be “housekeeping”, i.e., constitutively required, such as GAPDH (glyceraldehyde 3-phosphate dehydrogenase), used as loading controls in Western blotting [110]. However, since their levels are increased upon cell-to-cell contact, alternative proteins such as β-tubulin or Hsp90 may be preferred as markers instead [110].

Cell density and cadherin engagement are important and underappreciated parameters in the assessment of Stat3 activity. As experiments have been invariably conducted with actively growing cells, i.e., at subconfluence, the seismic changes occurring upon cell-to-cell adhesion may have been missed. This offers an explanation to previous puzzling findings from our lab and others where sparsely growing Src-transformed cells had very low p-Jak or Stat3-ptyr705 levels, unless they happened to grow in clumps i.e., adhering to each other [87].

## Data Availability

Data of experiments performed after 2013 are available in the supplementary data section of ref [48]. Unfortunately, older Western blotting experiments were performed using film and by students that have graduated and left the lab long time ago, at a time when preservation of raw data after a paper is published was not a requirement. The PI recently retired and had to clean her lab and old films were discarded.
